# Reframing stigma in Tourette syndrome: an updated scoping review

**DOI:** 10.1007/s00787-023-02332-3

**Published:** 2023-12-30

**Authors:** Kelly Pring, Melina Malli, Brandy W. Hardy, Stephen R. Rapp, Eric A. Storch, Jonathan W. Mink, Jaclyn M. Martindale

**Affiliations:** 1 Wake Forest University School of Medicine, Winston-Salem, NC USA; 2 Institute of Population Ageing, University of Oxford, Oxford, UK; 3 Department of Psychology and Behavioral Medicine, Wake Forest University School of Medicine, Winston-Salem, NC USA; 4 Menninger Department of Psychiatry and Behavioral Sciences, Baylor College of Medicine, Houston, TX USA; 5 Pittsford, NY USA; 6 Department of Neurology, Wake Forest University School of Medicine, North Carolina, Winston-Salem, NC USA

**Keywords:** Stigma, Tourette syndrome, Social–ecological, Discrimination, Tic, Scoping review

## Abstract

Persistent tic disorders (PTD) such as Tourette’s syndrome (TS) are common childhood-onset neurodevelopmental disorders. Stigmatization of individuals with these disorders remains an ongoing problem. The purpose of this scoping review is to serve as an updated review of the research regarding stigmatization in youth with PTD since the publication of the original systematic review about this topic in 2016. The electronic databases Embase, Web of Science, PubMed, PsycINFO, and CINAHL were searched. Of the 4751 initial articles screened after removing duplications, 47 studies met the inclusion criteria. The studies were examined under the social–ecological stigmatization model, which helps categorize stigmatization into individual, interpersonal, community, and structural levels and serves as a broader definition of stigmatization than the previous systematic review. On the individual level, youth with PTD had lower self-esteem than peers, often leading to fear of future stigmatization, avoidant behaviors, and self-stigmatization. They also experienced higher rates of bullying and other forms of abuse than peers at the interpersonal level. At the community level, youth with PTD faced discriminatory environments in school and work and limited availability of community services and healthcare access. At the structural level, knowledge about PTD was limited in the general population, often about the limited portrayals of PTD in the media. We hope that the broader scope of this review serves to help inform future efforts to decrease the stigmatization faced by this group.

**Supplementary Information:**

The online version contains supplementary material available at 10.1007/s00787-023-02332-3.

## Introduction

Persistent tic disorders (PTD), including Tourette syndrome (TS), are neurodevelopmental disorders clinically defined by multiple motor tics, vocal tics, or a combination for at least 1 year. TS affects 0.52–0.77% of children [1]. More than half of children who meet TS criteria may go undiagnosed. Tics begin gradually in early school age and peak in the peri-pubertal period [2–4]. Most tics improve through adolescence; however, persistent moderate-to-severe or worsening tics in adulthood can occur [4–6]. Co-occurring conditions, such as attention-deficit hyperactivity disorder (ADHD) [7], obsessive–compulsive disorder (OCD) [3], and anxiety [8], occur in 85% of individuals with TS.

For many chronic conditions, stigma has declined dramatically due to patient advocacy and activism strides. However, TS is often a visible and audible disorder that remains highly stigmatized [9–11]. Indeed, TS remains the second most judged chronic condition (behind migraine) in American media and news outlets between 1990 and 2018 [9]. Misconceptions, primarily related to complex tics such as coprolalia (obscene words), continue to be perpetuated and misunderstood by the public [11]. Youth with TS experience many stressors related to their diagnosis, including stereotypes, negative interactions, and opportunity loss. Stigmatization in the context of TS can lead to social rejection [12], avoidant behaviors [13], self-stigma, lower quality of life (QoL) [14], increased suicidality [15–17], worsened mental health [18], and tics persistence [5].

Additionally, individuals with TS already have more adverse general [19] and mental health outcomes [10, 18, 20, 21] yet inadequate accessibility to existing behavioral treatments [22] and limited non-pharmacological interventions focused on adapting to and coping with their TS [22–24]. There remains a poor understanding of why this stigmatization continues and how to address it best [12, 25]. Building upon previous work [11], our primary goal is to understand how stigma impacts youth with TS. However, our conceptual framework reframed the definition of stigma from prior work creating a broader scope of impact.

## Defining stigma and stigmatization

**Fig. 1:**
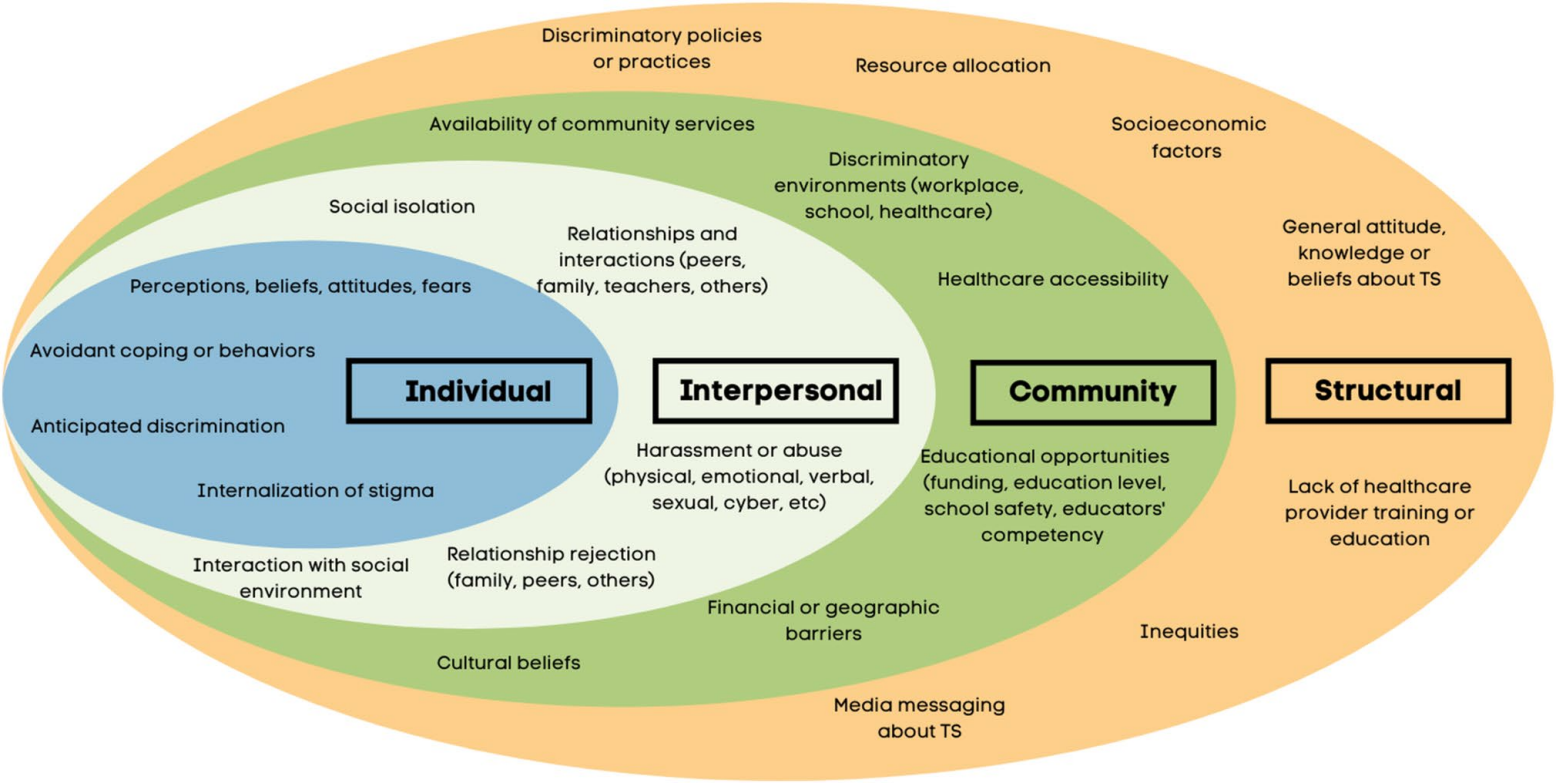
Social–ecological model of stigmatization in Tourette syndrome

The most widely cited definition of stigma is Goffman’s “an attribute that is deeply discrediting,” which “reduces the bearer from a whole and usual person to a tainted, discounted one” [26]. The link between the label of stigma and negative stereotypes [27] is argued as the rationale for believing a person is fundamentally different (‘us’ vs. ‘them’) [28]. A justification is then constructed for *stigmatizing* others [28]. Stigmatization refers to the act of being labeled, set apart, and linked to undesirable characteristics. Stigmatization can be overt, such as blatant discrimination, or more discrete, such as microaggressions or subtle biases, often leading to exclusion, mistreatment, or reduced opportunities. However, stigmatization is entirely contingent on social–ecological factors that allow the mark of stigma in the first place [28]. A similar understanding of stigmatization in HIV/AIDS [29, 30], mental illness [31], and transgender individuals [32] has been accomplished through the lens of the social–ecological model (SEM). Combining the SEM-based approach (Fig. 1) with Goffman’s definition of the mark of stigma can more thoroughly outline the factors leading to stigmatization in TS.

*Individual* stigmatization encompasses the affected individual’s fears, perceptions, beliefs, and attitudes [28, 30, 33]. Each influences how individuals cope. These experiences can lead to embodying negative stereotypes called internalization of stigma (*self-stigma*), affecting their self-efficacy, self-esteem, and self-concept. *Self-efficacy* refers to an individual’s belief in their ability to succeed in specific behaviors or actions, influencing motivation to initiate or preserve in the face of challenges. *Self-esteem* refers to the individual’s perception of their worth, value, and competence. *Self-concept* encompasses a comprehensive understanding of oneself, including various roles, attributes, abilities, and identities that a person identifies with.

*Interpersonal* stigmatization includes when individuals with TS interact with others and their environment. Most literature focuses on bullying, but a broader abuse scope must be included. These negative experiences interfere with daily activities in their social environment and can lead to social isolation.

The *community* level incorporates both organizational and community environments. Insurance availability and coverage, financial and geographic barriers, work and childcare coverage, and transportation accessibility impact healthcare accessibility and availability. Stigmatization also occurs within cultural, workplace, educational, and healthcare systems.

Lastly, *structural* stigmatization includes discriminatory policies and practices. These can be influenced by general beliefs about TS, inaccurate media portrayals, socioeconomic factors, and inequities. More broadly, government investment, or lack thereof, in resources, such as research funding, national organizations, support groups, educational initiatives, etc., influence the availability of services to individuals with TS. Importantly, this includes training or supporting the healthcare systems, including educating medical providers regarding the complexities of evaluating and managing TS.

The SEM allows a comprehensive evaluation of the stigmatization individuals face with TS. Successful interventions to combat stigmatization must consider a multifaceted approach [28, 31, 32]. The present scoping review serves as an updated review on stigmatization in TS through an SEM-based approach [11]. Our review aims to help as a broader look at stigmatization faced by those with TS, adding the community and systemic definitions of stigma to our inclusion criteria compared to previous work. By having a broader view of the definition of stigma, we hope that future efforts toward combating the stigma these individuals face can be more thoroughly informed.

## Relevance and implications of the updated scoping review

The first systematic review exploring stigma concerning TS was published in 2015. Despite clear evidence that stigma impairs well-being and mental health, the authors highlighted the lack of research exploring the experiences of individuals subject to TS stigma, the limited studies about public attitudes that stigmatize youths with TS, and the lack of work examining bias against people with TS among different cultural groups. The reviewed studies also highlighted methodological limitations, particularly small sample sizes, convenient sampling, and inappropriate measures. The authors called for more research in this understudied area to assess the nature of public and self-stigma, its prevalence, and the impact it may cause, and research in different contexts and cultures. Over this time, there have been many articles published on the topic. Guided by Arksey and O’Malley’s framework for scoping reviews [34], our overall objective is to investigate how the concept of stigma impacts youth with TS or PTD within the context of each SEM level. The concept of stigma in youth was chosen for this scoping review to align with prior work [11].

We had several sub-aims for the scoping review. First, to review individual stigma and why those with PTD develop self-stigmatizing thoughts and behaviors, such as avoidant behaviors, concealment of tics, and internalization of stigma. Second, to understand interpersonal stigma, including why individuals with PTD are at increased risk of social exclusion, verbal and physical abuse, and family and peer rejection. Third, to evaluate community stigma, including workplace, healthcare, and educational discrimination, faced by those with PTD. Fourth, to understand how larger structural systems, such as discriminatory policies and practices, health care access barriers, and cultural inequality disproportionately affect those with PTD. Lastly, to explore structural stigma through public attitudes and knowledge toward those with PTD and how this may contribute to the interpersonal stigma they face.

## Methods

### Study eligibility criteria

While the inclusion and exclusion criteria were mirrored from the prior systematic review, using the SEM broadens the definition to include the impact of community and structural stigmatization on individuals with TS.

To evaluate the concept of stigma, articles were included if they assessed (1) the youth’s perspective of stigma; (2) caregiver’s perspective of stigma; (3) retrospective reports of childhood experiences of stigma and discrimination by adults with TS; (4) self-stigma in youth with TS; and (5) courtesy or affiliate stigma in caregivers. To investigate how the concept of stigma impacts youth with TS or PTD within the context of each SEM level articles were included if they evaluated (1) Individual stigma; (2) interpersonal stigma; (3) community stigma; (4) structural stigma as defined by Fig. 1.

Articles were excluded if they evaluated (1) only adults’ experiences of stigma; (2) only measured post-intervention attitudes and behaviors toward individuals with TS without a pre-intervention assessment; (3) the impact of COVID-19; (4) functional tics or functional neurological disorder; and (5) certain types of evidence sources including Gray literature, non-English language literature, book chapters, reviews, posters, abstracts, and dissertations.

### Information sources

A search of the electronic databases Embase, Web of Science, PubMed, PsycINFO, and CINAHL was conducted on May 11, 2023. These searches yielded a total of 7206 studies before deduplication. The scoping review of the available literature was performed according to the Preferred Reporting Items of Systematic Reviews and Meta-analysis extension for Scoping Reviews (PRISMA-ScR) [35].

### Search strategy

The foundation of the search strategy for this review was derived from the 2016 systematic review [11]. The original search strategy was modified to include additional keywords to capture the relevant population, concept, and context studies. The results were limited to humans, the English language, and publication years 2015–2023. Study-type limits were not applied. The final strategy in Online Resource 1 was designed by a medical librarian in PubMed and translated to the other databases.

### Data management and collection process

All identified articles were uploaded into Covidence© systematic review software [36]. Titles and abstracts were independently reviewed for eligibility for full-text review by two researchers full-text review. Conflicts were discussed by all KP, JMM, and MM for consensus. Articles included for full-text review were independently reviewed by two researchers (KP and JMM) and evaluated for eligibility according to the inclusionary and exclusionary criteria. Conflicts were discussed and reviewed by KP, JMM, and MM for consensus.

### Data extraction

For each article, the following characteristics were extracted: first author, publication year, country of publication, study design, methodology, sample size, age of study population, study measures utilized, which level of SEM was addressed, and how based on the study conclusions.

### Study risk of bias assessment

A critical appraisal of the methodological quality of the included studies was not performed as it was not relevant to the aims of our scoping review. Studies that met inclusion criteria were not excluded based on study design.

## Results

### Included studies

**Fig. 2:**
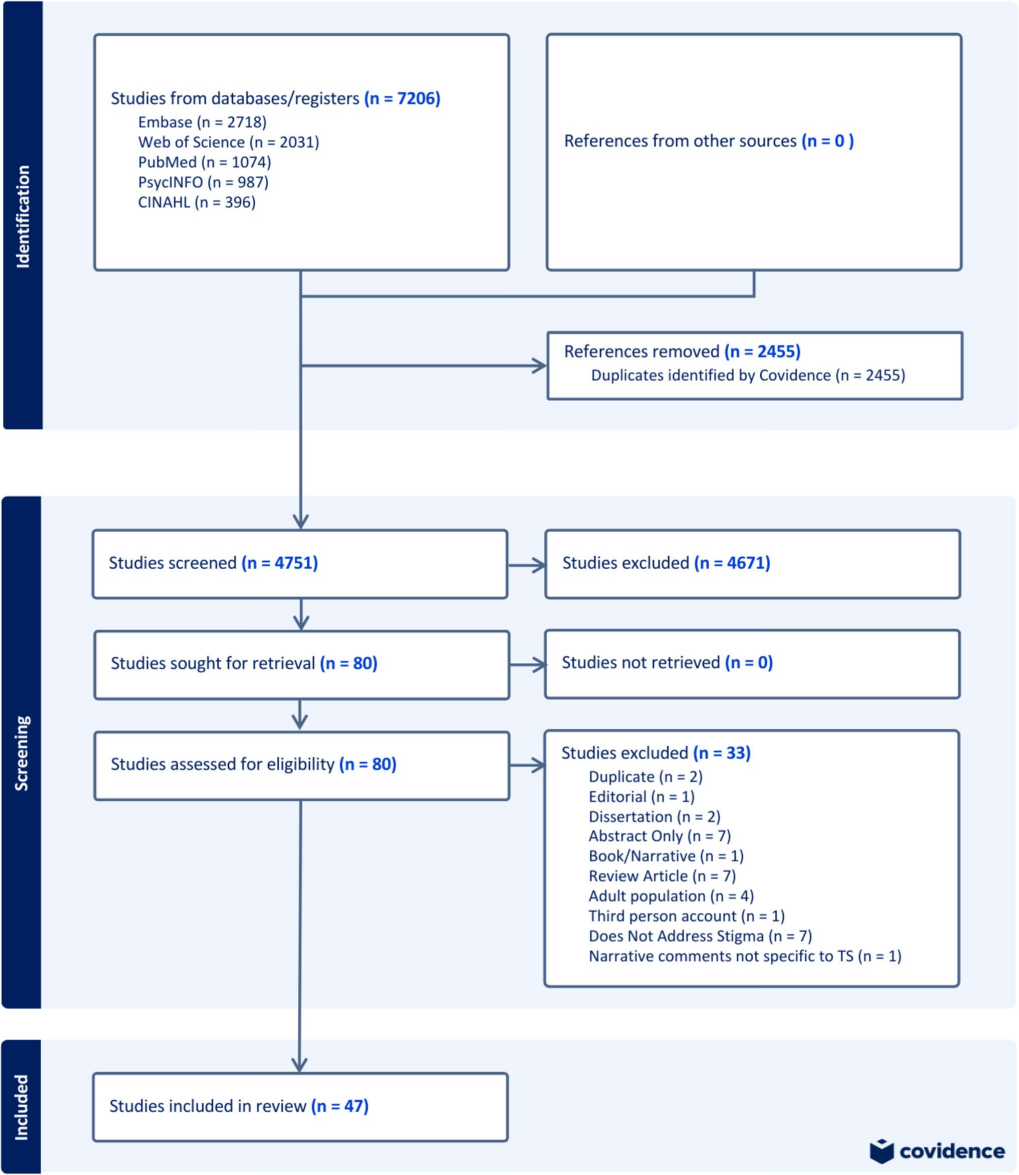
PRISMA-ScR chart of included studies

The database searches yielded 7206 results, with 4751 results for the title and abstract review after removal of duplicates. A PRISMA chart is included in Fig. 2. After a full-text review, 47 articles achieved consensus for inclusion.

### Overall results

Overall, a diverse range of studies was included (Table 1). The majority of studies were conducted in the United Kingdom (UK), 30% (14/47), and in the United States (US), 28% (13/47). Most study designs were cross-sectional studies, 43% (20/47), and with mixed-methods research, 19% (9/47). In the reviewed studies, 43% (20/47) focused on youth with TS, 28% (13/47) on the parents or caregivers of youth with TS, and 15% (7/47) general public or healthcare professionals. The most commonly addressed SEM levels were the interpersonal 64% (30/47) and the structural 62% (29/47).

**Table 1:** Overview of results (*n* = 47)

Countries included in studies	*n*	%
United Kingdom	14	30
United States	13	28
Europe^a^	7	15
Australia	6	13
Canada	4	9
China	5	11
Other countries^b^	8	17
Study designs		
Cross-sectional study	20	43
Mixed-methods study	9	19
Qualitative research	8	17
Prevalence study	3	6
Other designs^c^	7	15
Population included in studies		
Youth with TS	20	43
Parents/caregivers of children with TS	13	28
General public (caregivers of youth or adults without TS, public opinion)	7	15
Healthcare professionals	7	15
Adult with TS	6	13
Young Adults with TS	3	6
Educators	2	4
Other^d^	1	2
Level of stigma addressed		
Interpersonal	30	64
Structural	29	62
Community	19	40
Individual	18	38

^a–d^Each had 1–2 studies and was combined

^a^Netherlands, France, Germany, Norway, Sweden

^b^Bali, East Africa, New Zealand, Saudi Arabia, Korea and “other”

^c^Cohort study, thematic analysis, Q-methodology, non-randomized experimental study, and longitudinal person-centered ethnography

^d^Community threads (text-mining study)

### Data synthesis

We grouped the studies by the study location, design, population, and level of stigmatization addressed. The number of studies that met the criteria was compared to the total number that met the inclusion criteria. The level of stigmatization addressed was determined using an SEM-based approach with themes derived from prior medical models of stigma [29–32, 37] and previously presented in Fig. 1. KP and JMM independently reviewed all study findings and categorized results according to the SEM theme they aligned with. Conflicts were discussed and reviewed for a consensus. Study findings were not exclusive to one category, and detailed data categorization is included in Online Resource 2. Qualitative and quantitative studies were not separated for a more cohesive literature review. A summary table of the included articles is in Table 2.

**Table 2:**
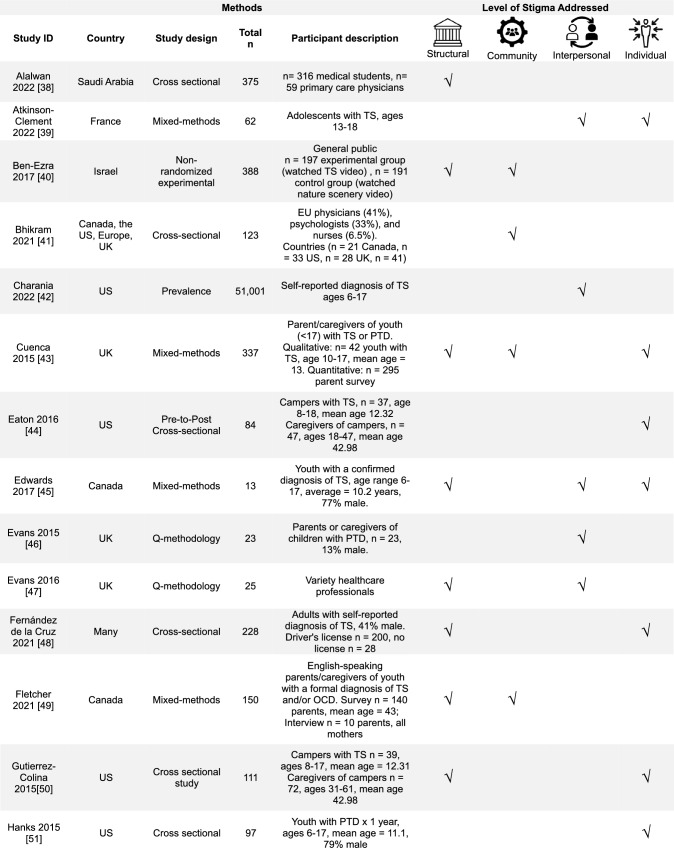

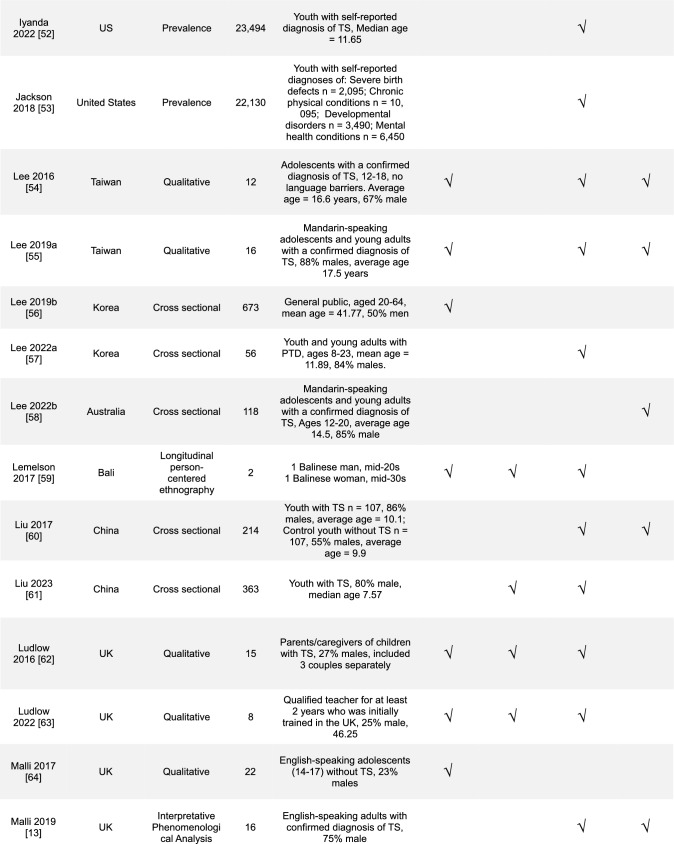

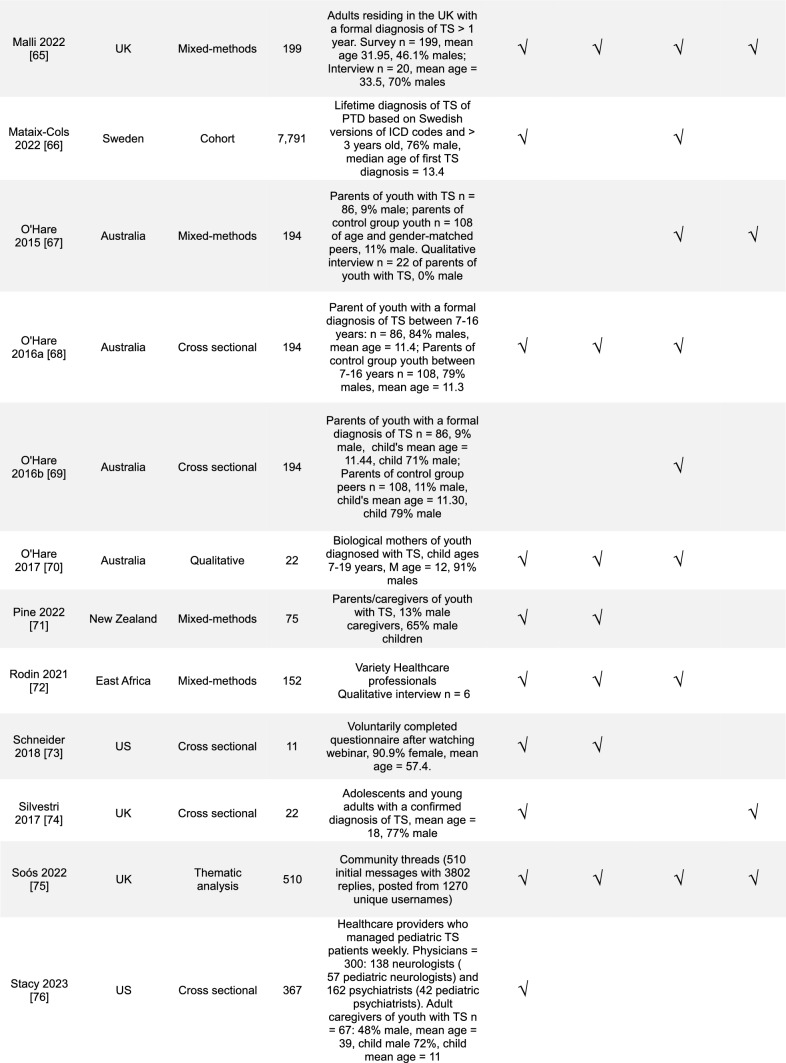

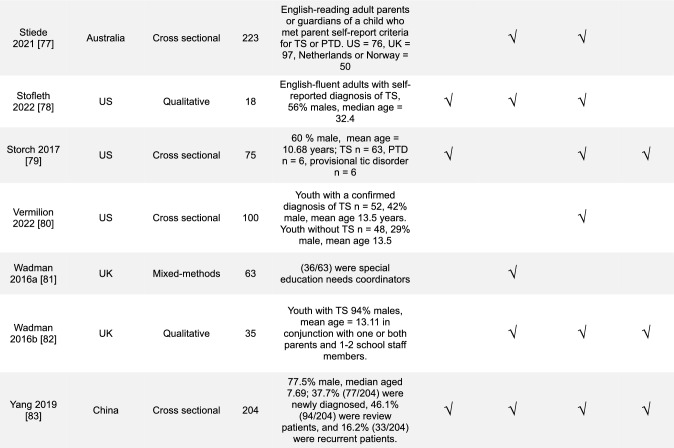
Table of studies

### Individual stigmatization

#### Perceptions, beliefs, attitudes, fears

Thirteen studies examined individuals’ perceptions, beliefs, attitudes, and fears of PTD [13, 39, 43–45, 50, 54, 55, 65, 67, 75, 82, 83]. Many feared being noticed, disruptive, or excluded by peers [39, 82]. They feared being mocked, teased, or rejected because of their tics [39, 67]. These fears increased with co-morbid conditions such as ADHD or OCD [39]. Youth with TS reported embarrassment and worry related to their experiences [45], which led to reduced motivation for peer interaction [67].

Many youths reported people did not understand TS, creating a sense of loneliness and segregation from normalcy [13, 54, 55]. Peer acceptance and maintaining social normalcy were significant motivators for individuals with PTD during all stages of life [67]. To maintain a sense of normalcy, individuals with TS often choose not to disclose their tics [75]. Youths avoid certain social situations out of fear that may interfere with peer relationships and lead to bullying or social exclusion [54]. Some adolescents give up school accommodations to feel more similar to their peers [54]. However, they also feared disturbing others in the classroom due to their tics during exams, which may have been relieved by these accommodations [82]. Similarly, adults hesitated to self-advocate for what they needed in their work lives to retain their sense of normalcy [65].

Interestingly, one study reported children had lower levels of self-perceived social anxiety than parental-reported social anxiety. This difference resulted from a youth’s poor self-awareness of their difficulties relating to other children [50] and lower social skills [67]. To further tease out this discrepancy, future studies should examine how social awareness changes over time in youth with TS to see if this relationship is more related to the child’s age or the TS itself.

Some individuals with TS saw methods and distractions helping to subdue tics as positive. In contrast, medications had more mixed opinions. Some felt medications create a sense of “otherness,” whereas others reported medications helped disguise tics in public, allowing youth to feel less self-conscious [43]. Medication adherence was low throughout childhood, with only 40% of youth having high medication adherence [83]. This was thought to be related to wanting to avoid the adverse effects and treatment by others for taking these medications.

As children age into adolescence, some grow their self-confidence and eradicate their negative emotions related to having TS [54]. Even further into adulthood, individuals could view their diagnosis as an opportunity for self-development, helping them find greater meaning in their lives [13]. Being surrounded by other peers with TS enabled youth to feel a sense of belonging and similarity to their peers [44]. Online support communities for individuals with TS were reported to be very helpful for many, providing belonging, community, encouragement, and resiliency [75]. However, many worry about the future, including how TS impacts career development, romantic relationships, and family planning [55]. In one study, over half of individuals with TS reported not applying for jobs or educational opportunities for fear of discrimination [65].

#### Avoidant coping or behaviors

Seven studies examined avoidant behaviors and coping in youth with TS [13, 51, 54, 65, 67, 79, 82]. Adolescents reported intentionally suppressing tics to avoid people’s attention in situations that may worsen their tics or interfere with peer relationships [13, 51, 54, 55, 67]. Some adolescents actively apologize or tolerate criticism to maintain peer relationships [54]. Social situations can invoke stress and anxiety in youth about their tics, and these emotions, in turn, worsen their tics [43]. This negative spiral emphasizes the importance of addressing co-morbid psychiatric disorders in youth with TS, such as anxiety or depression, to help both stress and tic severity.

Efforts to conceal their tics to maintain social relationships can be physically and mentally exhausting [13, 65]. Fear of peer rejection and difficulty spending long periods with friends suppressing tics can reduce peer interaction motivation [67]. Some individuals, adolescents, and adults fear others’ reactions to their tics in public so intensely that they withdraw and self-isolate socially [13, 54]. More severe tic-related impairment and avoidant coping strategies were associated with lower self-concept in youth with TS [51].

Several studies found that avoidant behaviors were associated with worse symptom severity and lower QoL [67]. Due to negative associations with avoidant coping, some studies mentioned intentional efforts to prevent youth with TS from avoiding activities. Families reported intentionally removing school absenteeism as an accommodation option because of their tics [79]. This illustrates the family’s desire to create structure and routine. On the contrary, poor understanding by the educational system of a child’s specific needs with TS can facilitate school avoidance [82].

#### Anticipated discrimination

Three studies examined anticipated discrimination faced by individuals with TS [48, 54, 65]. For adolescents, it was clear that they recognized the stigmatization they experienced because of their TS, putting them at a higher risk of anticipated discrimination than their younger counterparts [55].

Individuals with TS often choose not to disclose their tics to maintain normalcy but avoid anticipated discrimination [48, 54, 65]. To avoid being labeled as different from peers, some youth may choose not to use educational accommodations or medical exceptions in favor of perceived social normalcy [54]. Individuals withheld their diagnosis or suppressed their tics while obtaining a driver’s license out of fear it would obstruct their ability to get a license [48]. This was partly to avoid judgment due to their TS [48]. This similarly occurred in the workplace, where people hesitated to ask for reasonable accommodations for their TS to maintain social normalcy, even though this is a legal requirement of their employers [65]. By requesting accommodations, individuals feared job insecurity and perceived liability by their employers and avoided hiring them in future [65].

Anticipated discrimination is a genuine and common phenomenon faced by individuals with TS. Higher levels of anticipated discrimination were associated with poorer QoL [65]. Also, somewhat unsurprisingly, anticipating and experiencing discrimination were positively correlated [65]. Accordingly, if an individual has experienced such discrimination, they may be more likely to recognize its existence and fear future similar encounters. Screening for experienced and anticipated discrimination in the clinical setting should be considered. In these studies, it is unclear if there is any confirmation bias, such as if the individuals are anticipating discrimination, they may characterize the reactions of others as discriminatory more often than not. Regardless, affected individuals can benefit from appropriate interventions and services to help decrease future stigmatization risk.

#### Internalization of stigma

Eight studies examined internalization of stigma, including self-stigma, self-efficacy, self-esteem, and self-concept [13, 44, 51, 55, 58, 60, 65, 74]. Throughout the literature, many reports of embodied negative stereotypes and societal attitudes are sometimes called the ‘why try’ effect [84]. Adolescents reported their bodies projecting “repulsive” images, being labeled as socially unacceptable, and leading to a negative spiral of lacking confidence [55]. They said they were trying to control their tics to conceal their socially unacceptable identity [13]. Many endorsed having internalized public attitudes about TS, resulting in self-stereotyping and self-discrimination [65]. One individual went as far as to agree with the general belief that he saw himself as inferior and as having a devalued social identity as a person with a disability [65]. When TS had taken centrality in their lives and defined their sense of self, these individuals communicated more disadvantages, anger, and, most importantly, that TS deprived them of normalcy [13]. This was not true of all individuals, as some reported having lived with their diagnosis longer and come to terms with it and did not perceive TS as self-defining. They accomplished this through social downward comparisons, focusing on others worse off than themselves. Some individuals also reported that TS provided self-development and self-reflection opportunities [13].

Acceptance of the diagnosis improves with finding a support community [44]. When able to spend time with other youth with TS, youths’ social self-competence was significantly higher, and attitudes toward having TS were more favorable. However, there were no significant changes regarding their general self-competence [44]. These favorable findings further emphasize the importance of support groups within the TS community. Support groups promote belonging, community, and resiliency. As mentioned, these types of support groups do not exist to the extent this community desires, so more work is needed.

Overall, it was found that youth with PTD had poorer self-concept than age-matched peers, with no significant differences between genders. This finding was partially mediated by tic severity and depressive symptoms, suggesting that more severe disease and co-morbid conditions further worsen self-concept. Tic-related impairment and avoidant coping strategies also moderated the youth’s overall self-concept [51]. Overall, having at least one co-occurring psychiatric diagnosis was associated with significantly lower self-concept [74].

Tic severity was also negatively correlated with self-esteem and social adjustment and associated with more signs of insecurity [58, 60]. Individuals with TS reported poorer self-esteem due to the rejection and victimization they had experienced [13].

### Interpersonal stigmatization

#### Relationships and interactions with peers, family, teachers, and others

The most extensive topic was relationships and interactions with others covered by 23 studies [13, 39, 45–47, 54, 55, 57, 59–63, 65, 67, 69, 70, 72, 75, 77, 78, 82, 83]. Significant themes that emerged included difficulty making and maintaining friendships, reactions of others toward tics, complex interactions at school and work, and the family unit, both affecting and being affected by TS. Overall, individuals felt that their tics limited them by interfering with everyday activities, social life, and relationships in general [45, 60, 69, 75].

Just under 40% of youth with TS reported difficulty making friends compared with 4% of age-matched peers [69]. This number increased with age, as 70% of adults with TS had trouble making or keeping friends at some point [65]. This may be due to higher rates of insecure peer attachment [69], social anxiety [67, 69], stigmatization [67], and discrimination [65]. Peer interactions range from curiosity, sympathy, and ridicule to blatant discrimination [45, 54, 55, 65]. These adverse peer problems moderated the youth’s physical functioning, such as participation in sports and activities, perhaps due to social anxiety and avoidance [69]. However, with acceptance and affirmation by peers, TS is no longer a barrier to making friends. These positive interactions facilitate increased self-confidence and self-identity [54, 55].

Interactions within the educational system also posed difficulties. Youth reported the presence of bias against them by some teachers and classmates [54, 82]. Culturally, parents reported more significant reactions to tics in schools in the UK than in the US, the Netherlands, or Norway [77]. Many reported adverse reactions to their tics by teachers and students in the classroom [13, 82]. Mainly, tics were dismissed as behavioral, leading to negative judgment by others [13]. Additionally, teachers felt it was necessary to address the impact of TS on the other children and their families in the classroom [63], which may not always be done appropriately or sensitively [78]. Similarly to peers, however, when acceptance and respect were present from teachers, this encouraged youth to perform better in the classroom [54].

In public, individuals described various reactions to tics similar to the above. Some reported being approached by strangers to be asked or demanded to stop ticcing, staring, or being asked if they were okay [78]. While the last example considered their well-being, there was frustration due to a lack of understanding of the involuntary nature of the tics.

The majority of studies focused on family relationships and courtesy stigma. Similarly to the above, youth described various reactions to their tics by family, from acceptance [45], tolerance and respect [54], criticism [57], blame [13], and being discredited [13]. The family environment, including parental perceptions, expectations, and behavior, impacts a youth’s well-being [39, 46, 47, 57, 61]. Parental attitudes, body language, acceptance, and validation are essential for the youth’s development of self-image and self-esteem [46]. A lack of awareness, understanding, or acceptance of TS can promote a sense of being different and devalued, facilitating a youth’s internalization of stigma [13, 55]. The family may even withdraw from social events out of embarrassment or fear of disruption or inappropriateness of the tics to others [62, 70, 72].

Families may have difficulty separating symptoms from childhood misbehavior [70], place unreasonable demands on the youth [13], overly criticize [57], or coach the youth to conceal their tics from others and relatives [55]. More involved parents were more prone to blame their children, with criticism and negatively expressed emotions, leading to lower QoL in those with PTD [57, 61]. However, parents who were not fully involved, democratic parenting style, and inharmonious family relationships were associated with lower QoL in various domains [61]. Youth with TS feel more controlled in the home environment, insecure, less engaged, witness more family arguments, and communicate less with the adults in their home than their peers. Significantly, family climate can impact the QoL more than the tic severity [57].

Parents endorsed the dramatic effect of the parent–child relationship on youth’s well-being [46]. They recognized that their thoughts and behaviors contribute considerably to the child’s self-image, emphasizing the importance of a nurturing environment and parental validation [46]. Healthcare professionals mirrored these views, endorsing the importance of acceptance and hope for youth with TS through play, warmth, and praise [47]. Most importantly, the caregiver-child relationship and QoL were associated with medication adherence, with increasing QoL associated with high adherence [83]. These findings further emphasize the importance of parenting interventions, helping to create a more inclusive, caring environment.

However, TS affects not only the individual but the family unit as well. For one individual, her sibling suffered *courtesy or affiliate stigma* due to her TS. The siblings became undesirable marriage partners by having TS in the family [59]. Several studies report increased caregiver burden in taking responsibility for other’s reactions to their child’s tics and taking charge of their child’s care as their primary caregiver, expert, educator, and advocate [62, 70]. Caregiver burden was present for those even with a supportive and available partner. Additionally, with the diagnosis of TS, caregivers reported grieving the loss of their “ideal” child and had trouble preserving optimism for a normal future for their child [70].

#### Relationship rejection

Nine studies examined relationship rejection [13, 54, 55, 57, 59, 70, 72, 75, 77, 78]. Individuals reported experiences of mockery, dislike, mistreatment, exclusion, or hidden away by peers and family members [13, 54, 55, 59, 70, 72, 75, 78]. Some attributed this rejection to their sense of “otherness” and peers not wanting to “socially contaminate” themselves through their association [54]. Other individuals explicitly reported social rejection by peers, not wanting to be seen in public with them because of their tics [59]. The social rejection also encompassed work and romantic relationships [75].

Due to fears of relationship rejection toward the child and the family, some parents endorsed concealing the diagnosis of their child’s TS to relatives [55, 72]. Other families rejected the individuals themselves because of their TS diagnosis. An extreme example includes a father reporting that ‘if his daughter could not be cured of her TS, he wished she would die’ [59]. Still, other families said that because of their children’s diagnosis, they also experienced social exclusion with the loss of meaningful relationships and reduced social support [70].

#### Harassment and abuse

Seventeen studies examined the harassment and abuse experienced by individuals with PTD [13, 42, 45, 52–55, 57, 59, 65–68, 75, 78, 80, 82]. Overall, around half of those with TS feel they have been stigmatized by their disorder [67, 68]. Most literature focuses on bullying [42, 45, 52, 53, 57, 75, 80, 82]; however, unfortunately, there is a much broader scope that needs to be included, such as verbal [54, 55, 59, 65, 75, 78], physical [13, 65, 66, 75, 78, 82], and sexual assault [66]. Perpetrators encompassed a wide range of individuals, including those with TS [42], peers [54, 55, 65, 78, 82], family members [57, 78], neighbors [59], teachers [65, 78], coworkers [65, 78], or the general public [65, 78].

Overall, bullying victimization was higher among children with TS (35–56.1%) than children without TS (21.6–29%) [42, 80]. Being in middle school was associated with the highest risk of bullying victimization compared to elementary- or high-school-aged youth [52, 80]. ADHD and anxiety were also independently associated with bullying victimization, which are common co-occurring conditions with TS [52, 53]. Multiple conditions increased the risk of bullying victimization [53], which was negatively correlated with total and vocal tic severity [57]. Bullying occurs by peers, teachers, family members, coworkers, and strangers [45, 65, 75, 78]. Some even reported repeated bullying over the years [78]. Surprisingly, bullying perpetration was also statistically higher among youth with TS (20.7%) than those without (6.0%). Youth with TS were more likely to be both a perpetrator and a victim than youth without TS, which the presence of co-occurring conditions may explain after adjusting for age and sex, but was also associated with more severe tics [42].

Verbal abuse and mockery are common forms of harassment reported [78]. Individuals said they were being openly mocked, ridiculed, the target of jokes or demeaning laughter, and accused of faking their tics to humiliate or devalue their experiences [54, 55, 59, 65, 75, 78, 82]. Culprits of these verbal remarks included peers, strangers, siblings, caregivers, neighbors, and teachers [55, 59, 65, 78, 82]. Some youth reported being told to stop ticcing by peers or strangers or to “shut up” [78, 82]. Three individuals described being openly ridiculed and embarrassed by teachers in class in a way that felt intentional [78]. Outside of school, some individuals reported being verbally victimized on public transport, sometimes causing them to avoid public transportation [65].

Others shared experiences of being stared at and physically victimized [78], which affected their sense of personal security and safety [65]. Caregivers and youth reported specific incidents where they had been physically victimized at school, most often by peers [13, 65, 75, 78, 82]. One individual recalled being pushed down the stairs at school as a child [78]. Unfortunately, individuals with PTD have a twofold increased risk of any violent, sexual, or nonsexual assault compared to the general population [66]. One individual said he was physically harassed on a bus, while another was punched publicly for a misunderstanding [78]. These experiences and the high prevalence of abuse toward individuals with TS emphasize the importance of screening individuals regularly so that appropriate interventions can be made.

#### Interaction with social environment

Interactions with the social environment were examined by nine studies [39, 45, 60, 62, 65, 69, 72, 75, 79]. The emerging themes were the impact of tics on school [39, 45, 75], social activities [45, 60, 69], and family experiences [62, 65, 79]. Tics were disruptive to school work [45]. Motor tic severity and tic control impacted school concentration, reading, and writing abilities [39, 75]. Self-image and fear of disturbing others also affected school performance [39].

Compared with controls, youth with TS engaged less in home, social, and school activities, which worsens with more severe tics [60]. This may be related to the interference of tics in social activities shared by many individuals with TS [75]. Tics were found to be distracting in sports and other leisure activities [45]. Negative peer interactions affected youths’ physical functioning, such as participation in sports, possibly related to increased social anxiety [69].

Parents also reported their child’s TS affecting social activities and family experiences to avoid unwanted attention [62]. Almost 50% of parents reported modifying their leisure activities or work schedule because of their child’s needs at least once a month [79]. They highlighted daily struggles such as being able to hold down a job due to difficulty finding appropriate childcare for complex needs [62]. Some experienced leaving public places, either by choice or being asked, due to their child’s tics [62, 65].

#### Social isolation

Social isolation was discussed in three studies [13, 70, 72]. Caregivers and adults reflected on how stigmatization led to self-isolation and social avoidance [13]. Adults reported stigmatization during childhood stemmed from being seen as different, victimized, and excluded [13]. Mothers reported social isolation due to their child’s TS [70]. This may partly be due to voluntary social avoidance out of embarrassment or fear of the social implications of the tics [72]. As a result of the social isolation, mothers reported losing relationships and decreased social support, having to focus on and surviving as a nuclear family [70].

### Community stigmatization

#### Financial or geographic barriers

Four studies explored how financial and geographic barriers impacted individuals with TS and their families [41, 49, 59, 62]. The cost and affordability of healthcare professionals are limiting factors to access and receive healthcare by individuals. Additionally, the cost of medications and behavioral therapies can be prohibitive and impact treatment adherence. Specialists are often located in urban settings, creating additional barriers to access and receiving necessary care, including time off of work, travel to, transportation, and childcare for healthcare appointments [49]. As discussed in other sections, this is intimately tied to structural determinants, including socioeconomic status. Additional financial implications of tics can include replacing broken items and furniture as a result of the tics [62].

#### Availability of community services

Five studies included the availability of community resources [41, 43, 49, 75, 81]. The overarching themes focused on the availability of medical services to support care delivery [41], resource information [43, 49, 81], and online support communities [75]. Telemedicine services and individual CBIT therapy were most commonly available in the US compared to other regions. Limitations in telemedicine availability were a potential barrier to implementing CBIT therapy in areas, such as the UK and the EU [41].

Overall, there is a lack of available resources for individuals with TS and their families [43, 49]. These referred to various support measures, including an overall general lack of information regarding the diagnosis, resources on how to cope with the diagnosis, and educational resources to share with the school. There needed to be a central resource for what information is available, creating a frustrating experience for the individuals or their caregivers [49]. Many felt their healthcare professional provided little information and thought it necessary to search for additional resources [43]. Even seeking help from outside agencies, such as Child and Adolescent Mental Health Services (CAMHS), for resource support was difficult [81].

One study reviewed online support communities as safe, inclusive, and accessible places to share, unload, and ask for information about the realities of TS. Unfortunately, one of the realities discussed is the experience of dealing with inadequate TS-related health care [75].

#### Healthcare accessibility

Five studies examined healthcare accessibility barriers for individuals with TS [41, 43, 49, 68, 70]. Most described the diagnostic process as prolonged, traumatic, and difficult to obtain specialist referrals [43, 70]. During this process, the individual or the caregiver was required to assume the role of the educator and the advocate for the professional they consulted with, many of whom were dismissive of their concerns [70].

Additional barriers included limited knowledgeable specialists, resulting in long wait times, misplaced referrals, sporadic appointments, and clinic cancelations [41, 43, 49]. Referrals were often triaged based on the order in which they were received rather than severity or urgency [41]. Additionally, many providers reported seeing more follow-up patients than new patients. Ultimately, these inefficiencies led to delays in diagnosis, care, and treatments [49, 68]. Adults with TS faced more accessibility barriers than youth [41]. As mentioned above, specialists’ location, cost of services, and affordability affect an individual’s ability to access and receive healthcare [41, 49].

Once healthcare was established, caregivers and youth felt they needed more information to be provided by their healthcare provider about tics or TS [43]. There were differences in treatment preferences among healthcare professionals and the need for standardized treatment recommendations regarding therapeutic approaches. Affordability of medications and behavioral therapies were cited as inhibiting factors [49, 59]. Similarly, there was limited accessibility to knowledgeable behavioral therapists, resulting in a similar layer of barriers to behavioral treatment [43]. Additionally, behavioral therapy encounters preconceived perceptions about time and effort commitment and the notion of therapy in general [43].

#### Discriminatory environments

Discriminatory settings were discussed within eight articles while highlighting the significant challenges individuals face with TS as they interact with their surroundings [49, 59, 62, 63, 65, 70, 78, 82]. Educational environments [49, 59, 62, 63, 65, 78, 82] were the most commonly discussed, unsurprising, as most studies focused on youth or caregiver perspectives. Fewer studies disclosed discriminatory behavior within public settings [65], healthcare [62, 65, 70], and employment [65, 78]. Overall, TS individuals with co-occurring conditions report significantly more enacted discrimination than those without [65].

Negative experiences within the educational environment made up many of the challenges reported. There was a general lack of understanding of TS by educators, and it was not viewed as a disability [65]. Teachers endorsed that they were not adequately trained, and some even noted that the topic of TS was marginalized [63]. As a result, many reported receiving punitive action or frank discrimination rather than accommodations or support within educational settings [65, 78].

Within the classroom, several articles reported unhelpful staff responses to tics, the punishment due to tics, especially if inappropriate behaviors or coprolalia, the youth with TS being marginalized, or TS not being viewed as a medical disorder [59, 62, 82]. More often, there were significant barriers to accessing and following educational accommodations in the educational setting. To a lesser degree, incidents of being kicked out or removed from the classroom due to the youth’s tics [78]. Some individuals even reported school and educational leadership were purposefully unsupportive due to the perceived burden of accommodating a child with TS [49].

Within the workplace, some individuals with TS reported being denied employment interviews or opportunities because of their TS [65]. Many expressed needing more accommodations concerning their TS within the workplace. These included examples such as being excluded from training opportunities or requiring more flexible work arrangements. Unfortunately, TS can ultimately affect employment status [65, 78]. Request for accommodations, the difficulties imposed upon others, and ignorance of the cause of the tics or behaviors were considered deciding factors in an individual’s employment termination [65, 78].

Less commonly, individuals report feeling dismissed or invalidated during the diagnostic process within healthcare settings [62, 70]. Individuals also reported discriminatory behavior on public transportation, being asked to leave public places due to misconceptions about the etiology of symptoms, and misattribution of behavior by police officers [65, 78].

#### Educational opportunities

Eleven studies assessed educational opportunities [40, 49, 61–63, 65, 68, 71, 73, 81, 82]. Schools often needed more knowledge and preparedness to accommodate the learning needs of students in the classroom, which resulted in youth with TS being unable to fulfill their full potential [49, 63, 75, 81, 82]. Caregivers became educators and advocates in the classroom to teach about TS as ways to address stigma and bullying [49]. Teachers acknowledge they lacked the professional training to understand and support individuals with TS within schools [63, 81]. Barriers include limited staff, inconsistency of teachers, unwillingness, and lack of funding, time, or space to provide the necessary accommodations [71, 81]. Thus, in some instances, they were not offered additional time in exams or extension of assignments, as TS was not perceived as a legitimate disability [49, 62, 65, 71, 82]. Additionally, many caregivers articulated dissatisfaction with the effort required to obtain accommodations, lack of communication with the school system, and poor follow-through with accommodations despite being agreed upon [62, 65, 68]. Caregivers and youth often expressed frustration about inadequate support in mainstream schools and reported being faced with non-inclusive school beliefs and culture [49, 61–63, 68, 71, 82]. This leads to downstream effects, such as school avoidance and refusal [62, 82].

Professionals also reported inadequate knowledge, understanding, and experience with TS [63, 73, 81]. The educational level of people around individuals with TS, such as parents, educators, and professionals, affected the accommodation people received [40] and, subsequently, the QoL [61]. Individuals with higher levels of education were more likely to stigmatize youth with TS [40].

#### Cultural beliefs

Studies also examined differences in cultural views toward youth with PTD, both within and among cultures [59, 72, 77]. Two studies discussed local cultural beliefs of explaining symptoms due to evil spirits, displeased ancestors, inflicted punishment, or a mysterious contagion leading to shunning [59, 72]. In the Ugandan community, a study of healthcare professionals showed that children would be taken for alternative treatments, such as prayer, religious healers, or spiritual healers, instead of seeking medical care. This resulted in a need for more exposure to Ugandan healthcare professionals to TS [72]. An ethnography case study in Bali described cultural beliefs adding emotional and social impact to the individual’s TS. Her family’s inability to afford her medication reinforced their beliefs that her tics could not be solved by medical means [59]. Among cultures, parents reported higher reactions to youths’ TS in the UK than in the US, Netherlands, and Norway, which may be related to higher stigmatizing attitudes [77].

### Structural stigmatization

#### Discriminatory policies and practices

Four studies assessed discriminatory policies and practices [48, 65, 66, 75]. Some youth with TS were encouraged to be removed from mainstream classroom settings without clear justifications or formal proceedings [65]. This form of unofficial exclusion is unlawful, yet unfortunately, it is not a unique scenario. Adolescents reported experiencing difficulty getting their driver’s license because of their TS. The specific policies were unclear, with 13% required to provide a doctor’s note or documentation regarding their TS. Some chose to conceal their tics or withhold their diagnosis due to the time and financial burden of obtaining medical exams required to certify them for driving. However, 5% of individuals were denied licenses because of their TS and 2.5% had their licenses revoked, with only one feeling the decision was fair [48]. These findings illustrate the need to clearly outline the policies for people with PTD obtaining driver’s licenses and improve driving instructors’ awareness of tics to remove unnecessary judgment toward these individuals.

Peers believed individuals with TS face workplace hiring discrimination as tics may hinder career choices, specifically jobs requiring more face-to-face interaction [64]. These concerns were also voiced by individuals with TS themselves [65]. However, online TS support communities conferred that policies were in place to safeguard against this discrimination [75]. Members emphasized the legal protection of TS as a disability in the workplace, “They have to accommodate it reasonably and can’t fire you for it, under the disability law” [75]. Policies are in place to prevent workplace discrimination, yet efforts are needed to increase awareness and ensure they are followed.

Lastly, in Sweden, individuals with TS or PTD had a threefold increased risk of violent criminal convictions. The relative risk of conviction for violent crime was higher in women with TS. The cumulative incidence of nonviolent crime conviction was 39% in individuals with PTD compared with only 18% in the general population. This separation begins in early adolescence, around the age of 13 years [66]. It remains to be seen why this discrepancy exists or if it remains true in countries outside of Sweden, illuminating an area needing future research efforts.

#### General attitudes, knowledge, or beliefs about TS

There were 17 studies [40, 45, 48, 54–56, 59, 62–65, 70, 72, 73, 76, 78] that examined general beliefs about TS by different groups of people, namely those with TS [45], family members of those with TS [62, 70, 72, 76], peers [54, 64], teachers [63], community members (including other parents, neighbors, or the general public) [40, 48, 55, 56, 65, 78]. The general lack of exposure and understanding of TS and PTD has been discussed in other sections.

There were several reports of inaccurate statements regarding TS symptoms and etiology. Tics were misattributed as faking [64], lying [78], voluntary [64], or misbehaviors. Many presumed the diagnosis of TS was associated with coprolalia [62]. In some scenarios, tics were misinterpreted as personal attacks [78]. Others felt due to the chaotic nature of the tics, a physical distance should be maintained for personal safety [64]. Most importantly, tics were perceived as socially unacceptable behavior and contributed to being seen as “others” [64, 65]. Peers shared concern about the negative social consequences of being associated with an individual with TS. Additionally, peers felt this would limit an individual with TS’ future options in life [64]. These misunderstandings lead to conflicted views on how peers feel toward youth with TS [64].

The etiology of TS was also inaccurately assumed [45, 56]. Age, gender, extroversion, and familiarity with TS played a role in etiological beliefs, according to a South Korean online survey [56]. Cultural beliefs are an essential consideration in these studies. Older generations had a poorer understanding of TS [56, 62], and one study showed that they were more likely to believe in dietary or environmental etiologies of TS [56]. In the same study, women had greater beliefs in parenting or the psychological etiology of TS [56].

With these reports, it is unsurprising that many individuals with TS feel poorly understood [48, 54, 55]. This contributes to caregiver burden and various downstream consequences, including misattribution of blame, social stigma, relationship breakdowns, minimizing maternal concerns about their child, inadequate school support, and delayed treatment or diagnosis [70, 72, 76]. Teachers also voiced concern these misconceptions limit the acceptance and implementation of behavioral accommodations in the school setting [63]. Both peers and teachers of those with TS reported a need for knowledge about the disorder and limitations in reliable sources to learn more [54, 63].

#### Lack of healthcare provider training or education

Ten studies examined healthcare provider knowledge of PTD [38, 43, 47, 49, 62, 65, 68, 72, 73, 76]. Overall, healthcare professionals lack the necessary knowledge to diagnose and manage PTD [38, 43, 47, 49]. This was most noted in general practitioners (non-specialists) [38, 43, 47, 49, 65, 72], but often was not specified as to whom the individuals were discussing. Only 20–50% of individuals felt their or their child’s provider had adequate knowledge about TS or PTD [68, 76]. In one study, 38% of parents agreed with the statement, “I know more about TS than the healthcare provider” [76]. Unfortunately, this was associated with variability in care, including misdiagnosis, delayed diagnosis, and delayed treatment [43, 49, 65]. Some of this may be due to a lack of exposure to TS patients [38, 72]. Many practitioners were not confident in diagnosing, differentiating, recommending interventions, or managing PTD [47, 72, 73]. Treatment would be deferred until referred to a specialist, leading to long wait lists and delayed care [49]. Specialists in Neurology and Psychiatry reported higher confidence in several aspects of TS management [76]. However, as mentioned above, minimization of symptoms and outdated beliefs [38, 72] regarding the etiology of tics continue to exist even within the healthcare system, contributing to parental frustration and dissatisfaction and perpetuating stigmatization [68].

Though consensus supports the need for improved healthcare provider education about PTD, these studies may need to consider external factors. Two studies occurred in countries where medical system structure, exposure, and cultural influences may play a role [38, 72]. On the contrary, the study examining specialist’s confidence levels occurred in the US [76]. Despite this, in all countries, parents expressed dissatisfaction with provider knowledge about PTD, suggesting that this confounder of medical system structure does not affect the perceived quality of care. However, it means that future research efforts need to compare the confidence levels of healthcare providers at various stages of training and specialization across countries.

#### Injustice

The term injustice is a unifying word for oppression, marginalization, discrimination, stigmatization, and racism. It encapsulates the idea of unfair treatment, bias, and the denial of rights or opportunities to specific individuals or groups based on various factors, such as race, ethnicity, gender, social status, or disability. As the scoping review focuses on TS, four studies reviewed aspects of injustice surrounding gender [59, 66, 74, 79]. In Bali, gender significantly influenced the experience of TS, mainly related to the reactions to the tics. Differences in expectations of how women were expected to act led to more severe adverse reactions toward the tics despite the individual’s tics not being particularly severe. Additionally, cultural marriage practices forbade her from marrying her partner from a lower caste, which offered the opportunity to relieve financial burden and social stigma while providing social support. Such an option would not have been an issue if she was a man [59]. In Sweden, women had a higher relative risk of violent crime convictions than men. Additionally, women were more likely to experience any assault (violent, nonviolent, sexual) than men [66]. The reason for the gender differences was unknown and required further evaluation. Two other studies found no gender differences between self-concept [74] and family accommodations [79] in youth with TS.

#### Socioeconomic factors

Five studies examined the impact of socioeconomic factors on PTD [50, 65, 74, 79, 83]. There were mixed results. Sociodemographic variables, including age, gender, education, ethnicity, and marital status, were not associated with differences in enacted discrimination [65] or self-concept [74]. Age also did not impact the level of family accommodation provided for youth with TS [79]. However, the youth’s age, caregiver’s age, and perceived QoL were significantly associated with medication adherence [83]. Medication adherence was higher in younger individuals. Adolescents with more autonomy choose not to continue with their medications because of side effects or perceived stigma related to the nature of the medicines used to treat PTD, leading to lower adherence rates [83].

Youth’s age and gender were also associated with TS-related symptoms, including depression, hyperactivity, and inattention. The caregiver’s age was associated with the youth’s self-report of OCD symptoms. Family income affected both youths with PTD and their parents. Lower family income was associated with greater fear of humiliation in youth and increased depression in caregivers [50]. Marital status also impacted social functioning and perceived QoL in youth with PTD.

#### Media messaging about TS

Five studies examined how media messaging shaped perceptions and contributed to stigmatization in TS [62–65, 75]. Teachers [63] and peers [64] admitted to constructing their assumptions about TS through the media. Media portrayals are often inaccurate, reinforcing misconceptions and stereotypes [62, 65]. The media commonly depicts TS as coprolalia and voluntary while omitting other relevant details [64, 65]. Individuals with TS face the repercussions, dealing with frequent misunderstandings with all they interact with [75].

#### Resource allocation

Only two studies examined how resource allocation led to insufficient research and funding of educational initiatives [47, 81]. Both studies cited that these areas need to be improved and have the appropriate infrastructure necessary to succeed. The lack of research into parenting interventions was a barrier to treatment for many families of children with TS [47]. Also, lack of funding limited the behavioral accommodations teachers could offer students with PTD [63]. The inadequate availability of many services to individuals with TS further supports the overarching theme that active effort is needed in these areas.

## Discussion

This scoping review aimed to provide an update on the evidence of stigmatization toward TS. Although the total number of studies investigating the stigma of TS is still low, results indicate that stigmatization can constitute a severe concern for youth affected by TS and their families, affecting multiple facets of their daily lives.

Since the last systematic review in 2015, the current review indicates that TS stigmatization persists and remains in educational settings and close interpersonal relationships. Beyond these, recent studies suggest that TS stigmatization exists in the media, healthcare settings, policies, and practices, indicating that TS stigmatization does not simply occur at micro-level interaction. Still, it is embedded in macro-structures and plays a pivotal role in the distribution of resources. These practices disadvantage individuals with TS as they face structural constraints without individual discrimination and contribute to diminished population opportunities, resources, and well-being.

Research into TS stigma has slowly evolved, although essential advancements have occurred since the 2015 literature review. More studies have explored the personal experiences of people with TS and caregivers. There has also been an increase in studies published outside Western societies documenting TS in various settings. However, we need more in-depth empirical research, including qualitative research (e.g., interviews with young people with TS and family members) to fully understand the nuanced and layered stigma and quantitative studies (e.g., surveys to quantify the extent of stigma among crucial role players), to comprehend the full extent, nature and underlying mechanisms of TS stigma before we can develop interventions that may combat and reduce stigma. A more precise understanding of the origins and constructs of the stigmatization of TS could better inform future stigma reduction policies and improve engagement, peer relationships, and outcomes. Future longitudinal studies are needed to examine the impact of TS stigma over time.

To create effective interventions to reduce stigma and discrimination, it is important to target all levels of society where discrimination can occur. Hence, interventions should not only target the general public or students but should be supplemented by system-level interventions that prevent acts of discrimination.

There are currently no tools designed to measure TS stigma. Measures used to date have been from the broad mental health field. However, since TS stigma is constructed and manifests uniquely “off the shelf,” measures may not necessarily be fit for purpose. Validation of relevant scales to measure stigma may advance this field of research. Thus, it has been ascertained that stigma assessment tools based upon a theoretical model may help to promote progress in understanding the formative factors underpinning stigma and factors that may help to diminish stigma [85]. Healthcare personnel should consider these findings when providing care for people with TS.

## Limitations

Unpublished studies and other formats of publications, such as dissertations, were not included in this review. Although well justified, this exclusion may underreport the TS stigma studies in the review. In all of the studies, the vast majority of participants hold multiple marginalized identities and may have difficulty attributing stigma to one specific identity. Intersectionality theory suggests that people who hold additional marginalized identities do not experience stigma additively. Still, the multiple identities produce new experiences that cannot simply be reduced to the original identities that went into them. It is, therefore, unclear to what extent they could untangle the effect of the different conditions [86]. No studies specifically delineated the impact of stigma in TS only compared to TS with co-occurring conditions. However, since the majority of individuals with TS are affected by comorbidities, one could argue that untangling the effects of the intersectional synergies is irrelevant. Lastly, although a range of countries were represented in this review, the exclusion of research published in languages other than English limits the generalizability of the findings to different national and cultural contexts.

## Supplementary Information

Below is the link to the electronic supplementary material.

Supplementary file1 (DOCX 15 KB)

Supplementary file2 (XLSX 50 KB)
