# Supplementary material for: Reframing stigma in Tourette syndrome: an updated scoping review
Source: Eur Child Adolesc Psychiatry. 2023 Dec 30;34(1):19–39. doi: 10.1007/s00787-023-02332-3 (PMC11805784; doi:10.1007/s00787-023-02332-3)
Supplement: Supplementary file 1 — Supplementary file1 (DOCX 15 KB) [file 787_2023_2332_MOESM1_ESM.docx]

Online Resource 1: Full search strategy

PubMed 5/11/23

| 1 | Attribution*[tw] OR Attitude*[tw] OR stigma*[tw] OR prejudice*[tw] OR mistreat*[tw] OR injustice[tw] OR victim*[tw] OR bias* [tw] OR inequit*[tw] OR inequalit*[tw] OR disparit*[tw] OR prejudic*[tw] OR cultural*[tw] OR appropriateness[tw] OR securit*[tw] OR judge*[tw] OR exclus*[tw] OR fair*[tw] OR heurist*[tw] OR perception*[tw] OR knowledge[tw] OR ignoran*[tw] OR misconception*[tw] OR peer*[tw] OR opinion*[tw] OR stereotyp*[tw] OR social*[tw] OR rejection*[tw] OR bully*[tw] OR victim*[tw] OR discrimina*[tw] OR teas*[tw] | 4029592 |
| --- | --- | --- |
| 2 | (touret*[tw] OR tic[tw] OR tics[tw]) | 14593 |
| 3 | (2015:2023[DP]) | 10887854 |
| 4 | NOT (animals [mh] NOT humans [mh]) | 5111115 |
| 5 | english[la] | 30743932 |
| 6 | Attribution*[tw] OR Attitude*[tw] OR stigma*[tw] OR prejudice*[tw] OR mistreat*[tw] OR injustice[tw] OR victim*[tw] OR bias* [tw] OR inequit*[tw] OR inequalit*[tw] OR disparit*[tw] OR prejudic*[tw] OR cultural*[tw] OR appropriateness[tw] OR securit*[tw] OR judge*[tw] OR exclus*[tw] OR fair*[tw] OR heurist*[tw] OR perception*[tw] OR knowledge[tw] OR ignoran*[tw] OR misconception*[tw] OR peer*[tw] OR opinion*[tw] OR stereotyp*[tw] OR social*[tw] OR rejection*[tw] OR bully*[tw] OR victim*[tw] OR discrimina*[tw] OR teas*[tw] AND (touret*[tw] OR tic[tw] OR tics[tw]) AND (2015:2023[DP]) AND english[la] NOT (animals [mh] NOT humans [mh]) | 1074 |
| 6 | 1 AND 2 AND 3 AND 4 AND 5 | 1074 |
